# Innovations in scholarly communication - global survey on research tool usage

**DOI:** 10.12688/f1000research.8414.1

**Published:** 2016-04-18

**Authors:** Bianca Kramer, Jeroen Bosman

**Affiliations:** 1 Utrecht University Library, Utrecht, Netherlands

**Keywords:** scholarly communication, research workflow, survey, innovation, tools

## Abstract

Many new websites and online tools have come into existence to support scholarly communication in all phases of the research workflow. To what extent researchers are using these and more traditional tools has been largely unknown. This 2015-2016 survey aimed to fill that gap. Its results may help decision making by stakeholders supporting researchers and may also help researchers wishing to reflect on their own online workflows. In addition, information on tools usage can inform studies of changing research workflows.

The online survey employed an open, non-probability sample. A largely self-selected group of 20663 researchers, librarians, editors, publishers and other groups involved in research took the survey, which was available in seven languages. The survey was open from May 10, 2015 to February 10, 2016. It captured information on tool usage for 17 research activities, stance towards open access and open science, and expectations of the most important development in scholarly communication. Respondents’ demographics included research roles, country of affiliation, research discipline and year of first publication.

## Introduction

Many websites and tools exist to support researchers in handling information in all phases of the research cycle. For the first time a multidisciplinary and multilingual survey, carried out in 2015–2016, details the usage of such tools. Insights from these data may help researchers and those that support them in their decisions to improve the efficiency, openness and reliability of research workflows. Anonymized data from the survey is available in both raw (multilingual) and cleaned (all-English) versions (Data availability; 1). Details on data collection and full description of the data is provided in this Data Note. 

## Setup of the survey

The survey includes four questions on demographics, 17 on tool usage (with pre-selected answer options and free-text answer), two on support of Open Access and Open Science (yes/no/don’t know), one open question on the expected most important development in scholarly communication (free-text answer), one (optional) question asking for an email address and one question asking whether participants would be willing to be contacted for follow-up research. See the Supplementary material for the full list of survey questions in all languages.

Questions on demographics asked about country of current or last affiliation, research discipline, research role and career stage. Country of affiliation and research discipline were included because there is indication of strong variation in tool usage and publication cultures across these parameters. Our classification of research discipline (seven categories) was based on the broad classification from Scopus, with some modifications:

- Physical sciences (which in Scopus includes mathematics) - from which we made Engineering & Technology (including computer science) into a separate category
- Life sciences
- Health sciences - which we renamed Medicine
- Social sciences - from which we made Arts & Humanities and Law into separate categories.

Research role (which included various academic roles, but also supporting roles such as publisher, librarian and funder) and career stage (proxied by using the year of first publication in six date ranges) were included to allow testing hypotheses on e.g. the innovation of workflows being dependent on the degree to which people are conditioned by traditions in research practices. In addition, data on demographics can serve to assess and correct for bias.

**Figure 1.**
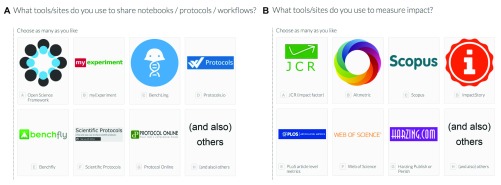
Examples of survey questions with preset answer options. **A**) Question on sharing notebooks/protocols/workflows. **B**) Question on measuring impact.

The bulk of the survey consisted of questions on tool usage for 17 activities in the research workflow (see Supplementary material and Table 4). These activities were selected from our database of research tools [ http://bit.ly/innoscholcomm-list], that distinguishes 30 research activities in seven phases of the research workflow and lists over 600 tools for these activities. The activities included in the survey were chosen for their overall importance (for example we included a question on writing tools but not on translation tools) and for their spread across the research workflow, covering discovery, analysis and writing as well as publication, outreach and assessment. For each of the 17 activities, the survey offered seven tools as preset answers and an eighth answer option to indicate use of any other tools ( Figure 1), followed by a question to specify those. The seven preset tools were chosen from the database of tools mentioned. In most cases we included 4–5 of the most well-known tools but also included 2–3 newer and smaller and in some cases even still experimental tools to stimulate respondents to also mention any less well-known tools they might use. Only in exceptional cases tools were offered as preset answer options in more than one question. Participants could skip any question (except demographic questions on research role, country of affiliation and research discipline) they felt did not apply to them, or were otherwise not willing to answer. Finally, people with a role supporting research were explicitly asked to base their answers to the questions on tools on what they would advise researchers to use.

All questions were entered into the cloud-based survey form software Typeform ( http://www.typeform.com). Typeform allows for ample use of graphics. These were used for all preset answers to tool usage questions. For these we used existing logos of tools and some self-made text logos. This made it very easy for respondents to recognize tools they used and enter most of their answers by simply clicking images.

## Distribution of the survey; sampling

The survey was live on the Typeform website for a 9-month period between May 10, 2015 and February 10, 2016. Responses submitted were stored by Typeform; a backup in csv format was made at regular intervals and stored on a university server.

The sample used was a fully open, self-selected, non-probability sample, meaning that the survey was open for anyone to take, with no systematic control on who took it. We used a hybrid of sampling methods, including snowball sampling and quota sampling. Distribution was targeted to researchers and people supporting research, both through direct and indirect distribution. Direct distribution included messages with the link to the survey on Twitter (e.g. in answer to people mentioning their paper/abstract/poster/manuscript got accepted), mailing lists, our own survey website, blog posts, including one on the widely read LSE Impact blog, a podcast interview on the Scholarly Kitchen website and during meetings the authors attended. Indirect distribution included that by 108 partners who distributed the survey among their constituency (either through a direct email message, inclusion in a newsletter or a message on the organisation’s website or intranet), in exchange for the anonymized data from that population. Of these, 65 organizations agreed to have their role disclosed. The 108 partners consisted of 76 universities (often through their libraries), 10 hospitals, 11 publishers and 11 other organizations. Some of these organizations also distributed our translations of the survey (see below). In addition, many individuals and organizations publicized the survey through various channels, e.g. through Twitter and other social media, in blogs and by inclusion in conference presentations. We did not specifically target students and know that many partners also did not do so.

**Figure 2.**
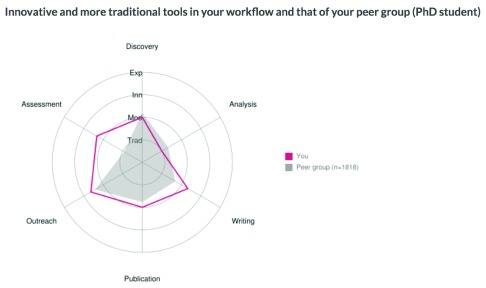
Example of automatic feedback received by survey participants. Classification: **Traditional tools (Trad)** - Add no functionality compared to print era, except online accessibility; **Modern tools (Mod)** - Use scale and linking possibilities of the internet to increase speed and efficiency; **Innovative tools (Inn)** - Actually change ‘the way it’s always been done’ – e.g. user-driven, different business models, changes in the sequence of research activities, shifting stakeholder roles; **Experimental tools (Exp)** - Represent radical change, with sometimes uncertain technologies and outcomes; still under development. Tools were scored on a scale of 1 (traditional) to 4 (experimental); the chart shows average scores per workflow phase. Tools mentioned as ‘others’ are not included at this stage.

We offered respondents no financial incentives or presents to stimulate take up. However, all respondents were offered the option to receive automatic feedback ( Figure 2) on how their choices of tools compared to those of their peer group (based on research roles entered). For this we used a dataflow from Typeform via Google Drive ( http://drive.google.com, for calculations and creating the graphs) to WordPress ( http://www.wordpress.com to publish the graphs). To transfer data between these tools we used Zapier ( http://www.zapier.com).

## Translation of the survey

To address cultural and language bias and simply to increase uptake in non-English language areas we had the survey translated into six world languages: Spanish, French, Chinese, Russian, Japanese and Arabic. These languages were selected based on observed underrepresentation of these language areas after four months of having the survey available only in English. However, this was done only after attaining initial success with attracting respondents to the survey and after getting requests for translation. Translations became available in the 6 ^th^ month (Spanish and French), the 7 ^th^ month (Chinese and Russian), the 8 ^th^ month (Japanese) and 9 ^th^ month (Arabic) of the survey period.

The survey was professionally translated, and reviewed by at least two native speakers (one researcher and one librarian). All questions and preselected answer options were kept identical across different language versions. However, in five of the six foreign language versions (the exception being Arabic) we included one additional question at the end of the survey on the use of tools targeting that specific language area. This was done to increase commitment, to stimulate respondents to also mention language-specific tools and to be able to check answers given here against tools mentioned as ‘others’ in the regular survey questions.

## Distribution of responses

In total, 20663 valid survey responses were received. Obvious spam responses (n=6) were removed from the data.

**Distribution channels** - Responses received could be traced back to distribution channels by way of a suffix attached to the survey URL ( Table 1). Although in absolute numbers the foreign language versions contributed only modestly to the overall response numbers ( Table 2), they were quite important to stimulate response from the respective language areas ( Figure 4).

**Table 1.** Survey responses by distribution channel.

Channel	Responses
Mailing lists	485
Partners: publishers	9070
Partners: universities & hospitals	6463
Partners: others	541
Survey website	2604
Twitter	1220
Social media other than Twitter	57
Other / unknown	223
**Total**	**20663**
Responses removed (spam)	6

**Table 2.** Survey responses by language version of the survey.

Language version of survey	Responses
English	17785
Spanish	1052
French	955
Russian	330
Chinese	265
Japanese	258
Arabic	18
**Total**	**20663**

**Country of current or last affiliation** - Partly helped by the translations we got a very broad response from across the globe with at least 1 response from 151 countries and at least 20 responses each from 64 countries ( Figure 4).

**Figure 3.**
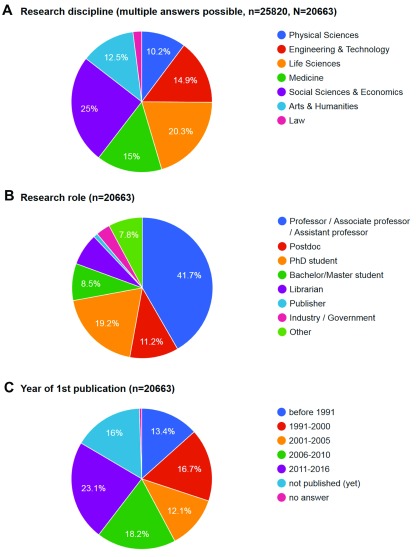
Demographic distributions of survey responses. **A**) Mentions of research discipline(s) (multiple answers possible, 25820 answers given, N=20663). **B**) Responses by research role (n=20663). **C**) Responses by year of first publication (n=20663).

**Research discipline** - The largest group of respondents was from social science and economics. Other disciplines were also well represented, with only law lagging ( Table 3, Figure 3A).

**Figure 4.**
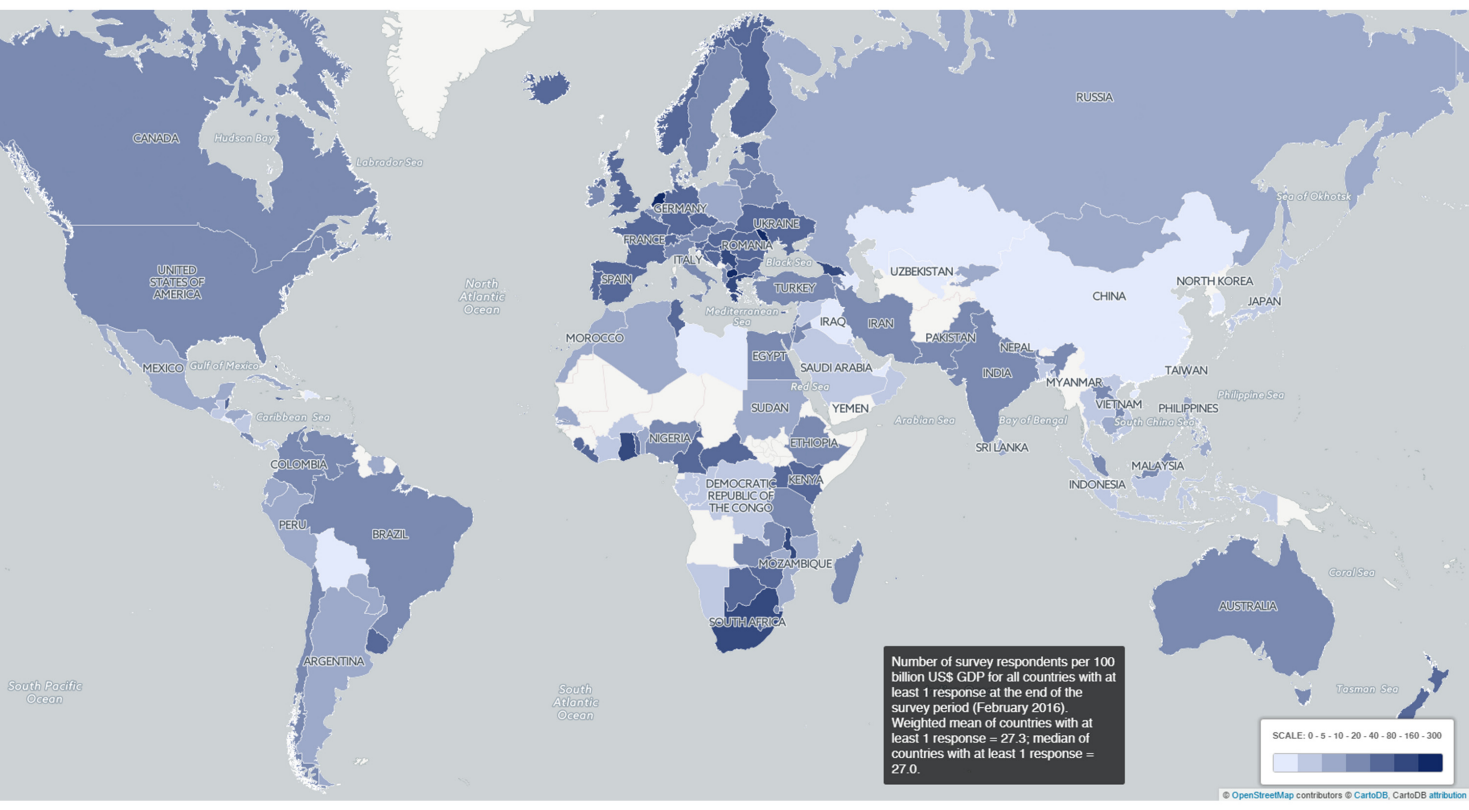
Survey response levels per 100 billion US$ GDP (2013). Number of survey responses per 100 billion US$ GDP for all countries; weighted mean of all countries with at least 1 response: 27.3, median: 27.0.

**Table 3.** Mentions of research discipline(s) (multiple answers possible, 25820 answers given, N=20663).

Research discipline	Mentions
Physical Sciences	2644
Engineering & Technology	3838
Life Sciences	5246
Medicine	3879
Social Sciences & Economics	6465
Arts & Humanities	3228
Law	520
**Total**	**25820**

**Research role** - The vast majority of respondents are from inside academia (from students to professors) ( Table 4, Figure 3C). Relatively few students responded, probably because many considered themselves not active researchers yet. Other groups are also much smaller, allowing for less detailed analysis.

**Table 4.** Survey responses by research role (n=20663).

Research role	Responses
Professor/Associate professor/ Assistant professor	8610
Postdoc	2312
PhD student	3974
Bachelor/Master student	1756
Librarian	1517
Publisher	199
Industry/Government	677
Other	1618
**Total**	**20663**

**Career stage** - Table 5 shows career stage of respondents carrying out research as measured by year of first publication ( Figure 3C). Interestingly there is a fairly even distribution, indicating interest in the topic of the survey across various ages and career stages. Please note that the answer ‘ *not published (yet)*’ may indicate that the respondent is in the beginning of a researcher’s career, but also that someone has a role in which publishing is not a primary task. To identify these separate populations, demographic data for career stage can be combined with those on research role.

**Table 5.** Survey responses by year of first publication (n=20663).

Year of 1 ^st^ publication	Responses
Before 1991	2763
1991–2000	3454
2001–2005	2505
2006–2010	3763
2011–2016	4763
Not published (yet)	3300
No answer	115
**Total**	**20663**

## Population, sample size & response rate estimation

With an open self-selected survey like this there is no fixed sample size and thus reporting response rates is not straightforward. However we have made estimations of the total number of people that has been targeted in our distribution efforts (1.4 million, Table 6). This number represents an upper limit as it does not account for overlap in populations reached through various modes of distribution. Based on this estimation, the overall response rate is 1.5%. We can also relate the number of responses to officially reported numbers of researchers (i.e. response compared with total target population) and look at response rates from specific partners that distributed the survey to a defined number of researchers (i.e. response of a subset of the population). This latter approach also allows for comparison of response rates across different modes of distribution. For instance, in cases where the survey was distributed via a mass mailing response varied between 1 and 10 percent, reached within less than a week. In cases where partners used an indirect message to an undefined set of people (e.g. through a message on intranet or on social media) very few responses were generated (typically a few dozen, even when the stated target group contained many thousands of people), and it often took months to reach that number.

**Table 6.** Population, sample size and response rate indicators.

	Size	Rate
Population size: worldwide number (head counts) of researchers, based on [ 2, p. 31]	7.8 M	
Sample size: estimation of total number of people targeted by survey distribution; breakdown: - Twitter, direct (@ tweets, estimated) - Twitter, indirect (general tweets, estimated) - Mailing lists (not deduplicated) - Others (blogs, meetings) (estimated) - Distribution by custom URL partners (estimated), among which: - - Universities - - Publishers - - Hospitals - - Others	~1.4 M 2700 8773 25799 7000 ~1.3 M 155921 1136401 6333 17033	~18% (=relative sample size)
Response size	20663	1.5% (= response rate)

## Completeness of the responses

Not all questions received answers from all respondents and not all answers were valid. Table 7 shows the number of answers per question and the number of valid answers (where applicable). Also shown are the number of respondents that indicated they used (also) other tools (or had another research role) than the ones mentioned as preset answer, and how many of those specified these other tools or research roles.

**Table 7.** Number of answers per survey question. # answers = total number of answers per survey question; # answers valid (*) = number of valid answers per survey question (where applicable); # answers yes (**) = number of respondents answering ‘yes’ per survey question (where applicable); # others = number of respondents that checked the ‘other’ option per survey question (where applicable); # others specified = number of respondents that specified ‘others’ as free text answers.

Question	# answers	# answers valid* or yes**	# others	# others specified
***Demographics***				
Research role	20663		1534	1531
Country	20663	20608*		
Discipline	20663			
Year of 1st publication	20548			
***Tool usage per activity***				
Search	20453		8009	7340
Alerts	20238		3479	2933
Access	16463		4900	4276
Read	20029		3584	3271
Analyze	18577		6876	6366
Share protocols/notebooks	7426		5015	3540
Write	20354		2354	2186
Reference management	16471		2908	2268
Share publications	15658		3477	2961
Share data/code	7516		3660	2239
Select journal	11901		3071	2277
Publish	15646		1931	1277
Share posters/presentations	7752		3219	1994
Outreach	11539		3899	2932
Researcher profiles	17374		1583	1239
Peer review	4783		2010	495
Measure impact	13213		1872	1304
Language-specific tools	2238		207	116
***Other questions***				
Most important development	12209	12060		
Support Open Access	19013			
Support Open Science	19157			
E-mail address	9562			
Can we contact you?	18464	10033**		

## Anonymization of the data

On our website and in the survey itself, we guaranteed participants only anonymized data would be shared. We anonymized the data by:

- Removing email addresses where given;
- Removing information on the specific custom URL through which the response was received;
- Generalizing research role specifications where traceable to specific persons (either directly or through combining with other information);
- Generalizing information given about the country of affiliation (sometimes much more detailed affiliations were given);
- Removing identifiable information from free text answers.

We had to be extra careful because we do not only share the full data, but also shared subsets containing just the data of respondents invited by the respective partners through the custom survey URLs. In cases where those partners were academic institutions or hospitals, they know the institutional affiliation of respondents in that subset, making possible identification from free text answers potentially more likely.

## Cleaning and harmonization of the data

For the cleaned dataset we harmonized free-text answers by correcting spelling (of e.g. country names and tool names), unifying acronyms and full names, and grouping similar answers that used different phrasing (e.g. “library databases” and “bibliographic databases”). For country of affiliation, we also replaced names of areas that constitute part of a country with the name of the country as a whole. For this we used the UN list of member and observer states. For instance, responses attributed to people from overseas areas of France and Britain simply got assigned the main country as country of affiliation. In the answers given as specification of other tools used for a certain activity, responses that contained identifying information and could not be generalized to a more generic tool name were categorized as “other”. Cases where respondents indicated they either use no specific tool for an activity or do not engage in the activity were removed as answers. As we chose not to let respondents specify reasons for not answering questions, these answers are conceptually no different from cases where respondents skipped a question altogether.

Both raw answers and cleaned/harmonized answers are available as separate datafiles, but identifying information is removed from raw answers to guarantee anonymity (see above).

## Reverse translation of foreign language answers

Reverse translation of answers given in languages other than English was initially done by using Google Translate. The use of automated translation was justified as most answers contained just simple text, e.g. names or descriptions of tools used. For the answers on the open question on expectations of the most important development in scholarly communication, translations provided by Google Translate were manually checked by the authors for French and Spanish, and in cases of doubt help from a native speaker with domain knowledge was requested. Free text answers to this question given in Chinese, Arabic, Russian and Japanese were also translated by a professional translation service. These translations were compared with the Google Translate texts and in cases of major discrepancies the translations were put before a native speaker with domain knowledge. In all cases, both the original answers and the most suitable translation are provided in the dataset, except where identifying information was removed from raw answers to guarantee anonymity (see above).

## Observed and expected biases in the data

Given the nature of the data collection we expect biases to be present in the data. The demographic data we collected can be used to both assess for biases (by comparing against known distributions within the target population) and overcome them, e.g. by zooming in during analyses. For instance, if the distribution over research roles seems not proportional, one could focus analysis on one group only. Where that is not viable raking is a statistical method that can be used to correct distributions, if the distribution in the overall population is known. Of course this only needs to be done if one suspects the variable at hand to be correlated with that distribution.

To check for regional bias we compared numbers of responses per country to the size of that country’s GDP ^4^, which we took as a crude proxy for the number of researchers. Figure 4 depicts that bias. Measured thus, the Netherlands and some other small European countries are represented far above average and many West-African and Central and Southeast Asian countries way below average or not at all. Given their large absolute sizes, the low levels of response in countries such as China and Korea are noteworthy.

Biases not directly related to the demographic parameters included in the survey will be harder to assess. For instance, we were unable to confirm whether there is bias along the degree to which people are interested in or concerned about scholarly communication issues.

## Data description, data storage and sharing

The total size of both the raw and cleaned versions of the data is 20663 records and 178 variables, of which 162 for the tools questions and 16 for demographics and other general questions. File format is csv. These files with supplementary material are bundled into one zipped citable data set with DOI identifier.

The measurement level of the majority of the data is nominal (tools used, affiliation, role, discipline), in a few cases ordinal (indication of support for Open Access and Open Science) and only once interval (year ranges for year of first publication).

For permanent storage, the anonymized data are deposited in Zenodo under a CC-0 license. In addition, raw data will be stored for up to five years on secure Utrecht University servers for further analysis, with email information in files separate from the rest of the data.

In addition, we have made the data available through an interactive dashboard on Silk ( http://dashboard101innovations.silk.co/) to enable quick visual exploration of the data.

## Consent

The research is subject to the code of conduct of the Dutch Association of Universities (VSNU) ^3^.

## Data availability

The data referenced by this article are under copyright with the following copyright statement: Copyright: © 2016 Kramer B and Bosman J

Data associated with the article are available under the terms of the Creative Commons Zero "No rights reserved" data waiver (CC0 1.0 Public domain dedication). 

*Zenodo*: Global survey on research tool usage, doi: https://dx.doi.org/10.5281/zenodo.49583 ^1^

## Supplementary material

- . Survey questionnaire text and graphics, in English, Spanish, French, Chinese, Japanese, Russian and Arabic.
