# Supplementary material for: Innovations in scholarly communication - global survey on research tool usage
Source: F1000Res. 2016 Apr 18;5:692. [Version 1] doi: 10.12688/f1000research.8414.1 (PMC4934514; doi:10.12688/f1000research.8414.1)
Supplement: Supplementary file 1 [file f1000research-5-9058-s0000.tgz › 300a78fa-e12e-4789-ab1f-4cb604012e9a.zip!/Surveys/101_Innovations_survey_Arabic.pdf]

الخصائص الديمغرافية والتخصصات 1

a. ما هو دورك في البحوث؟ *

- أستاذ / أستاذ مشارك / أستاذ مساعد
- باحث ما بعد الدكتوراه
- طالب دكتوراه
- طالب بكالوريوس / طالب ماجستير
- أمين مكتبة
- الناشر
- الصناعة / الحكومة
- غير ذلك

b. ما هو بلدك الذي تنتمي إليه حاليا أو بلد انتماءك الأخير؟ *

c. ما هو التخصص/التخصصات التي تعمل فيها؟ *

- العلوم الفيزيائية
- الهندسة والتكنولوجيا
- علوم الحياة
- الطب
- العلوم الاجتماعية والاقتصاد
- القانون
- الآداب والعلوم الإنسانية

d. متى نشرت أول مادة خاصة بك؟

- لم أنشر بعد
- 2011-2016
- 2006-2010
- 2001-2005
- 1991-2000
- قبل 1991

في الجزء التالي من الاستبيان، نطلب منك تحديد أي الأدوات/المواقع التي تستخدمهاللأنشطة البحثية المختلفة

لا تتردد في ذكر الأدوات التي تستخدمها والتي قد تكون خاصة ببلدك أو لغتك.

يمكنك دائما تخطي الإجابة عن سؤال إذا كان النشاط البحثي لا ينطبق عليك أو إذا لمتكن ترغب في الإجابة عليه.

إذا لم تكن باحثا نشطا (ولكن أمين مكتبة، ناشر، الممول الخ)، يرجى الإشارة إلىالأدوات التي توصي بها.

اكتشاف 2

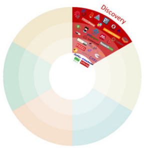

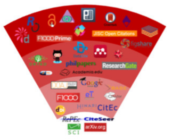

a. ما هي الأدوات/ المواقع التي تستخدمها للبحث عن الأدبيات/البيانات/ ما إلى ذلك؟

- Google Scholar
- Web of
- Scopus
- Mendeley
- WorldCat
- PubMed
- Paperity
- (وأيضا) غيرها — وأيضا (آخرين)

b. ما هي الأدوات/المواقع التي تستخدمها للوصول إلى الأدبيات وما إلى ذلك؟

وصول مؤسساتي (الوصول من خلال مؤسستي أو مكتبتي)

- [ ] وصول مؤسساتي

Purchase

- [ ] الدفع عن كل إطلاع

ResearchGate

- [ ] ResearchGate

research4life
HINARI · AGORA · OARE · ARDI

- [ ] Research4Life

- [ ] Open Access

deepdyve

- [ ] Deepdyve

- [ ] أرسل بريدا إلكترونيا

(وأيضا) غيرها

- [ ] (وأيضا) غيرها

c. ما هي الأدوات/المواقع التي تستخدمها للحصول على تنبيهات / التوصيات؟

Google scholar

- [ ] Google Scholar

Journal TOCs

- [ ] JournalTOCs

BrowZine

- [ ] Browzine

- [ ] Mendeley

F1000 Prime

- [ ] F1000 Prime

sparrho

- [ ] Sparrho

ResearchGate

- [ ] ResearchGate

(وأيضا) غيرها

- [ ] (وأيضا) غيرها

d. ما هي الأدوات/المواقع التي تستخدمها لقراءة / الاطلاع/ كتابة الحواشي؟

بالاطلاع بطريقة لغة توصيف النص التشعبي (عرض نسخة لغة توصيف النص التشعبي عبر الإنترنت)

(وأيضا) غيرها

- [ ] Acrobat
- [ ] بالاطلاع بطريقة لغة
- [ ] iAnnotate
- [ ] ReadCube
- [ ] UtopiaDocs
- [ ] Mendeley
- [ ] Hypothes.is
- [ ] وأيضا) غيرها)

التحليل 3

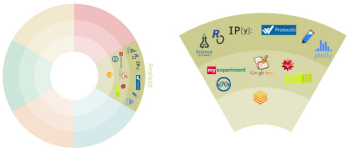

a. ما هي الأدوات/المواقع التي تستخدمها لتحليل البيانات/النصوص وما إلى ذلك؟

(وأيضا) غيرها

- [ ] R
- [ ] SPSS
- [ ] Matlab
- [ ] Excel
- [ ] iPython
- [ ] ROpenSci
- [ ] DHbox
- [ ] وأيضا) غيرها)

b. ما هي الأدوات/المواقع التي تستخدمها لتبادل المذكرات / البروتوكولات / سير العمل؟

- [ ] Open Science
- [ ] myExperiment
- [ ] BenchLing
- [ ] Protocols.io
- [ ] Benchfly
- [ ] Scientific
- [ ] Protocol
- [ ] (وأيضا) غيرها

الكتابة 4

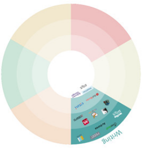

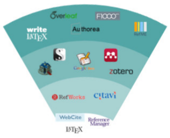

a. ما هي الأدوات/المواقع التي تستخدمها لكتابة/إعداد نص؟

- [ ] Word
- [ ] Google
- [ ] Authorea
- [ ] LaTeX
- [ ] Scrivener
- [ ] Overleaf
- [ ] Scalar
- [ ] (وأيضا) غيرها

b. ما هي الأدوات/المواقع التي تستخدمها لإدارة المراجع؟

- EndNote
- Zotero
- RefWorks
- Mendeley
- Papers
- REfME
- Citavi
- (وأيضا) غيرها

(وأيضا) غيرها

النشر 5

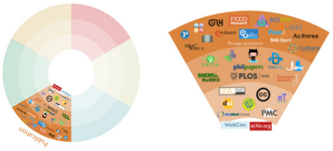

a. ما هي الأدوات/المواقع التي تستخدمها لأرشفة/ تبادل المنشورات؟

- arXiv
- PubMed
- المستودع المؤسساتي
- bioRxiv
- أشارك أوراق العمل

المستودع المؤسساتي

أشارك أوراق العمل

(وأيضا) غيرها

- ResearchGate
- SSRN
- (وأيضا) غيرها

b. ما هي الأدوات/المواقع التي تستخدمها لأرشفة/تبادل البيانات وترميزها؟

- GitHub
- Figshare
- Zenodo
- Dryad
- Dataverse
- Pangaea
- BitBucket
- (وأيضا) غيرها
  (وأيضا) غيرها

c. ما هي الأدوات/المواقع التي تستخدمها لاتخاذ قرار بشأن المجلة التي تريد تسليم مادتك لها؟

- JCR (impact
- DOAJ
- Scopus
- Sherpa Romeo
- QOAM
- SCImago
- Journalysis
- (وأيضا) غيرها
  (وأيضا) غيرها

d. ما هي الأدوات/المواقع التي تستخدمها للنشر؟

- مجلة موضوعية (ناشر)
- مجلة موضوعية (ناشر)
- مجلة ميجا (ناشر)
- مجلة ميجا (ناشر)
- مجلة بيانات
- Winnower
- F1000Research
- (وأيضا) غيرها

---

6 الانتشار

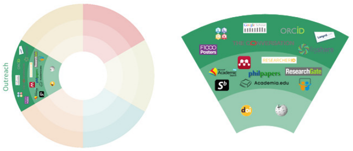

a. ما هي الأدوات/المواقع التي تستخدمها لأرشفة/تبادل الملصقات الإعلانية والعروض التقديمية؟

- Speakerdeck
- Slideshare
- F1000Posters
- ScienceOpen
- Figshare
-
-
- (وأيضا) غيرها

Zenodo	Vimeo	(وأيضا) غيرها

b. ما هي الأدوات/المواقع التي تستخدمها للتحدث عن البحوث الخاصة بك خارج المجال الأكاديمي؟

☐ Wikipedia	☐ ResearchBloggi	☐ Wordpress	☐ Kudos	☐ FameLab
☐ Pint of Science	☐ Twitter	(وأيضا) غيرها<br>☐ (وأيضا) غيرها		

c. ما هي الملفات الشخصية للباحثين التي تستخدمها؟

☐ Google Scholar	☐ ResearchGate	☐ ORCID	☐ Academia.edu	☐ ResearcherID
صفحة الملف الشخصي في المؤسسة<br>☐ صفحة الملف	☐ My Science	(وأيضا) غيرها<br>☐ (وأيضا) غيرها		

التقييم 7

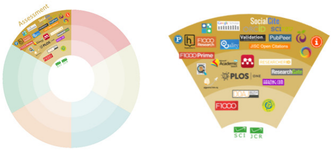

a. ما هي الأدوات/المواقع التي تستخدمها لمراجعة الأقران التي تتجاوز ما تنظمه المجلات؟

This is about commenting and peer review, pre- as well as post-publication

- [ ] Peerage of
- [ ] Publons
- [ ] PubMed
- [ ] PubPeer
- [ ] PaperCritic
- [ ] RubriQ
- [ ] وأيضا) غيرها)
- [ ] (and also)

(وأيضا) غيرها

b. ما هي الأدوات/المواقع التي تستخدمها لقياس التأثير؟

- [ ] JCR (impact
- [ ] Altmetric
- [ ] Scopus
- [ ] ImpactStory
- [ ] PLoS article

(وأيضا) غيرها

8 ثلاثة أسئلة عامة اخيرة حول التواصل العلمي

a. برأيك، ماذا أهم تطور في مجال التواصل العلمي في السنوات القادمة؟

b. هل تؤيد هدف الوصول المفتوح؟

أ: نعم ب: لا جـ: لا أعرف

c. هل تؤيد هدف العلوم المفتوحة؟

(i.e. openly creating, sharing and assessing research, wherever viable)

أ: نعم ب: لا جـ: لا أعرف

9 يمكنك أيضا الإشارة ما إذا كان يمكن الاتصال بك لمواصلة مناقشة كيف يقرر الباحثون الأدوات التي يستخدمونها للتواصل العلميلن يتم عرض بريدك الإل

a. إدخال عنوان البريد الإلكتروني الخاص بك هنا (إذا كنت ترغب في ذلك)

b. هل يمكننا الاتصال بك لمناقشة سير عمل التواصل العلمي؟

Yes No

إرسال
