# Supplementary material for: Innovations in scholarly communication - global survey on research tool usage
Source: F1000Res. 2016 Apr 18;5:692. [Version 1] doi: 10.12688/f1000research.8414.1 (PMC4934514; doi:10.12688/f1000research.8414.1)
Supplement: Supplementary file 1 [file f1000research-5-9058-s0000.tgz › 300a78fa-e12e-4789-ab1f-4cb604012e9a.zip!/Surveys/101_Innovations_survey_Chinese.pdf]

1 背景特征与学术领域问题

a. 您在研究工作中的职衔是什么？ *

- ○ 教授/副教授/助理教授
- ○ 博士后
- ○ 博士生
- ○ 学士/硕士生
- ○ 图书管理员
- ○ 出版人
- ○ 行业人士/政府部门
- ○ 其他

b. 您当前（或之前）所属机构的所在国家是哪个？ *

____

c. 您从事哪个（哪些）学科的研究？ *

- ☐ 理科
- ☐ 工程技术
- ☐ 生命科学
- ☐ 医学
- ☐ 社会学与经济学
- ☐ 法学
- ☐ 人文学科

d. 您第一次发表学术刊物是在哪一年？

○ 尚未发表 ○ 2011-2016 ○ 2006-2010 ○ 2001-2005 ○ 1991-2000
○ 1991 年之前

本问卷调查的下一个部分需要您说明您用于各种科研活动的工具/设施。

尽管填写只有您所在国有的工具，或是只有您的语言版本的工具。

若某项科研活动不适用，或您不愿回答，可随时跳过问题。

如果您不是研究人员（而是图书管理员、出版人、赞助人等），请指出您推荐的工具。

---

2 探索

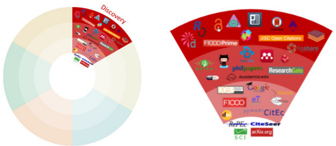

a. 您用什么工具/设施来查找文献/数据等资料？

- ☐ Google Scholar
- ☐ Web of
- ☐ Scopus
- ☐ Mendeley
- ☐ WorldCat
- ☐ PubMed
- ☐ Paperity
- ☐ （以及）其它

b. 您用什么工具/设施来获取文献等资料？

机构资料库<br>☐<br>机构渠道（通	Purchase<br>☐<br>在出版商平台	ResearchGate<br>☐<br>ResearchGate	research4life<br>☐<br>Research4Life	☐<br>Open Access
deepdyve<br>☐<br>Deepdyve	@<br>☐<br>给作者发送电	（以及）<br>其它<br>☐<br>（以及）其它		

c. 您用什么工具/设施来收取提示/推荐讯息？

Google scholar<br>☐<br>Google Scholar	Journal TOCs<br>☐<br>JournalTOCs	BrowZine<br>☐<br>Browzine	☐<br>Mendeley	F1000 Prime<br>☐<br>F1000 Prime
sparrho<br>☐<br>Sparrho	ResearchGate<br>☐<br>ResearchGate	（以及）<br>其它<br>☐<br>（以及）其它		

d. 您用什么工具/设施来阅读/查看/备注资料？

Acrobat

在线查看 HTML 版

用 HTML 查看

iAnnotate

ReadCube

UtopiaDocs

Mendeley

Hypothes.is

（以及）其它

（以及）其它

3 分析

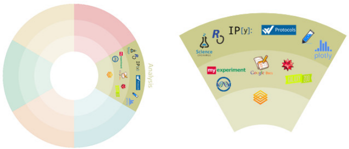

a. 您用什么工具/设施来分析数据/文本等资料？

R

SPSS

Matlab

Excel

iPython

ROpenSci

DHbox

（以及）其它

（以及）其它

b. 您用什么工具/设施来共享指导手册/原则协议/工作流程？

- Open Science
- myExperiment
- BenchLing
- Protocols.io
- Benchfly
- Scientific
- Protocol
- （以及）其它

（以及）

其它

4 编撰

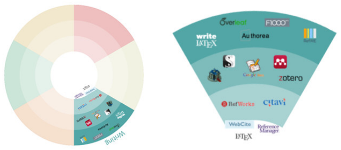

a. 您用什么工具/设施来编撰/准备文稿？

- Word
- Google
- Authorea
- LaTeX
- Scrivener
- Overleaf
- Scalar
- （以及）其它

（以及）

其它

b. 您用什么工具/设施来管理参考文献？

- EndNote
- Zotero
- RefWorks
- Mendeley
- Papers
- REfME
- Citavi
- （以及）其它

（以及）其它

5 出版

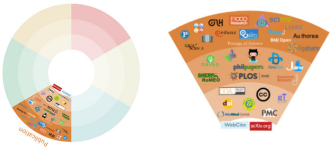

a. 您用什么工具/设施来归档/共享出版物？

- arXiv
- PubMed
- 机构资料库
- bioRxiv
- 共享工作论文

机构资料库

共享工作论文

（以及）其它

- ResearchGate
- SSRN
- （以及）其它

b. 您用什么工具/设施来归档/共享数据和代码？

- GitHub
- Figshare
- Zenodo
- Dryad
- Dataverse
- Pangaea
- BitBucket
- （以及）其它

c. 您用什么工具/设施来判断哪一份期刊适合投递您的文稿？

- JCR (影响因子)
- DOAJ
- Scopus
- Sherpa Romeo
- QOAM
- SCImago
- Journalysis
- （以及）其它

d. 您用什么工具/设施来发表？

- [ ] 专题期刊（传
- [ ] 专题期刊（开
- [ ] 综合期刊（传
- [ ] 综合期刊（开
- [ ] 数字期刊
- [ ] Winnower
- [ ] F1000Research
- [ ] （以及）其它

---

6 推广

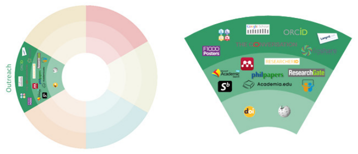

a. 您用什么工具/设施来归档/共享宣传海报和演示文稿？

- [ ] Speakerdeck
- [ ] Slideshare
- [ ] F1000Posters
- [ ] ScienceOpen
- [ ] Figshare
- [ ]
- [ ]
- [ ] （以及）其它

Zenodo	Vimeo	（以及）其它

b. 您用什么工具/设施来向学术界以外的大众介绍您的研究？

- ☐ Wikipedia
- ☐ ResearchBloggi
- ☐ Wordpress
- ☐ Kudos
- ☐ FameLab
- ☐ Pint of Science
- ☐ Twitter
- （以及）其它 ☐ （以及）其它

c. 您使用哪种研究人员档案管理工具？

- ☐ Google Scholar
- ☐ ResearchGate
- ☐ ORCID
- ☐ Academia.edu
- ☐ ResearcherID
- 机构自有的档案管理页面 ☐ 机构自有的档
- ☐ My Science
- （以及）其它 ☐ （以及）其它

7 评估

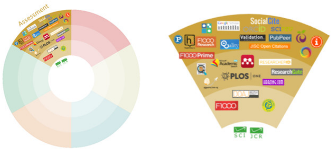

a. 您用什么工具/设施在期刊开展的同行评审之外进行同行评审？

涉及发表前后的评价与同行评审

- ☐ Peerage of
- ☐ Publons
- ☐ PubMed
- ☐ PubPeer
- ☐ PaperCritic
- ☐ RubriQ
- ☐ Academic
- ☐ （以及）其它

b. 您用什么工具/设施来评测影响力？

- ☐ JCR (影响因子)
- ☐ Altmetric
- ☐ Scopus
- ☐ ImpactStory
- ☐ PLoS article

（以及）

其它

8 你用哪一个中国的工具？

- 小木虫
- 微博
- 百度学术
- 中国知网
- 理文编辑
- 万方数据
- 华艺在线图书
- （以及）其它

（以及）

其它

9 最后三个关于学术交流的问题

a. 在今后的几年里，您认为什么在学术交流领域里会有最重大的进展？

b. 您是否支持以开放获取为目标 (Open Access)?

- 是
- 否
- 不知道

c. 您是否支持以开放科学为目标(Open Science)?

(即在任何可行的情况下开放式地创造，分享以及评估科学)

是 否 不知道

---

10 如果您希望收取自己的答复概览，请在下方输入您的电邮地址。亦请注明您是否愿意我们联系您，与您进一步讨论研究人员应如何选择学术交流的工具。我们不会披露，亦不会出于其它目的使用

a. 请在此输入您的电邮地址（如果您愿意）

b. 我们能否联系你进一步讨论学术交流的流程？

是 否

传
