# Supplementary material for: Innovations in scholarly communication - global survey on research tool usage
Source: F1000Res. 2016 Apr 18;5:692. [Version 1] doi: 10.12688/f1000research.8414.1 (PMC4934514; doi:10.12688/f1000research.8414.1)
Supplement: Supplementary file 1 [file f1000research-5-9058-s0000.tgz › 300a78fa-e12e-4789-ab1f-4cb604012e9a.zip!/Surveys/101_Innovations_survey_English.pdf]

1 Demographic & Discipline(s)

a. What is your research role? *

- Professor / Associate professor / Assistant professor
- Postdoc
- PhD student
- Bachelor/Master student
- Librarian
- Publisher
- Industry / Government
- Other

b. What is the country of your current (or last) affiliation? *

c. What discipline(s) are you working in? *

- Physical Sciences
- Engineering & Technology
- Life Sciences
- Medicine
- Social Sciences & Economics
- Law
- Arts & Humanities

d. From which year dates your first scholarly publication?

○ I haven't published (yet) ○ 2011-2016 ○ 2006-2010 ○ 2001-2005
○ 1991-2000 ○ before 1991

In the next part of the survey, we ask you to identify which tools/sites you use for various research activities.

You can always **skip** a question if that particular research activity does not apply to you.

If you are not an active researcher (but a librarian, publisher, funder etc), please indicate which tools you **recommend**.

---

2 Discovery

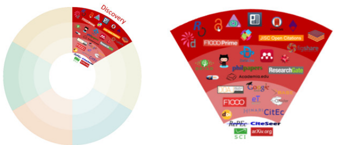

a. What tools/sites do you use to search literature / data / etc.?

- ☐ Google Scholar
- ☐ Web of
- ☐ Scopus
- ☐ Mendeley
- ☐ WorldCat
- ☐ PubMed
- ☐ Paperity
- (and also) others ☐ (and also)

b. What tools/sites do you use to get access to literature etc.?

access through my institution or library ☐ Institutional	Purchase ☐ Pay per view	☐ ResearchGate	☐ Research4Life	☐ Open Access
☐ Deepdyve	☐ E-mail the	(and also) others ☐ (and also)		

c. What tools/sites do you use to get alerts / recommendations?

☐ Google Scholar	☐ JournalTOCs	☐ Browzine	☐ Mendeley	☐ F1000 Prime
☐ Sparrho	☐ ResearchGate	(and also) others ☐ (and also)		

d. What tools/sites do you use to read / view / annotate?

viewing HTML version online

- [ ] Acrobat
- [ ] using HTML
- [ ] iAnnotate
- [ ] ReadCube
- [ ] UtopiaDocs

(and also) others

- [ ] Mendeley
- [ ] Hypothes.is
- [ ] (and also)

---

3 Analysis

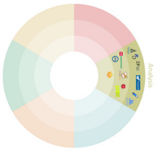
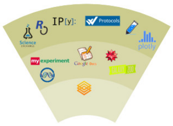

a. What tools/sites do you use to analyze data / texts etc.?

- [ ] R
- [ ] SPSS
- [ ] Matlab
- [ ] Excel
- [ ] iPython

(and also) others

- [ ] ROpenSci
- [ ] DHbox
- [ ] (and also)

b. What tools/sites do you use to share notebooks / protocols / workflows?

- [ ] Open Science
- [ ] myExperiment
- [ ] BenchLing
- [ ] Protocols.io
- [ ] Benchfly
- [ ] Scientific
- [ ] Protocol
- [ ] (and also) others — (and also)

4 Writing

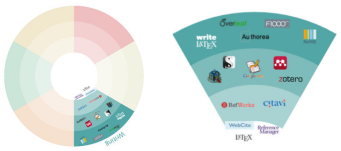

a. What tools/sites do you use to write / prepare your manuscript?

- [ ] Word
- [ ] Google
- [ ] Authorea
- [ ] LaTeX
- [ ] Scrivener
- [ ] Overleaf
- [ ] Scalar
- [ ] (and also) others — (and also)

b. What tools/sites do you use for reference management?

- [ ] EndNote
- [ ] Zotero
- [ ] RefWorks
- [ ] Mendeley
- [ ] Papers
- [ ] REfME
- [ ] Citavi
- [ ] (and also) others — (and also)

5 Publication

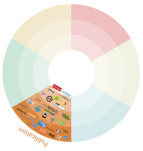

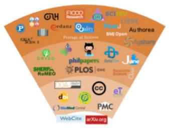

a. What tools/sites do you use to archive/share publications?

- [ ] arXiv
- [ ] PubMed
- [ ] Institutional Repository — Institutional
- [ ] bioRxiv
- [ ] [Working Papers] — I share working
- ResearchGate
- SSRN
- (and also) others

- ResearchGate
- SSRN
- (and also)

b. What tools/sites do you use to archive/share data & code?

- GitHub
- Figshare
- Zenodo
- Dryad
- Dataverse
- Pangaea
- BitBucket
- (and also) others — (and also)

c. What tools/sites do you use to decide which journal to submit your manuscript to?

- JCR (impact
- DOAJ
- Scopus
- Sherpa Romeo
- QOAM
- SCImago
- Journalysis
- (and also) others — (and also)

d. What tools/sites do you use to publish?

- [ ] Topical journal
- [ ] Topical journal
- [ ] Megajournal
- [ ] Megajournal
- [ ] Data journal
- [ ] Winnower
- [ ] F1000Research
- [ ] (and also)

(and also) others

6 Outreach

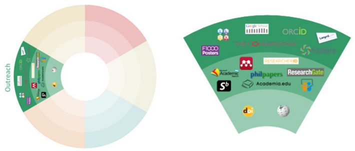

a. What tools/sites do you use to archive/share posters & presentations?

- [ ] Speakerdeck
- [ ] Slideshare
- [ ] F1000Posters
- [ ] ScienceOpen
- [ ] Figshare

(and also) others

☐ ☐ ☐

b. What tools/sites do you use to tell about your research outside academia?

☐ Wikipedia	☐ ResearchBloggi	☐ Wordpress	☐ Kudos	☐ FameLab
☐ Pint of Science	☐ Twitter	(and also) others ☐ (and also)		

c. What researcher profiles do you use?

☐ Google Scholar	☐ ResearchGate	☐ ORCID	☐ Academia.edu	☐ ResearcherID
profile page at own institution ☐ Profile page at	☐ My Science	(and also) others ☐ (and also)		

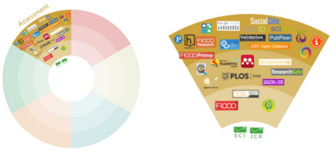

a. What tools/sites do you use for peer review beyond that organized by journals?

This is about commenting and peer review, pre- as well as post-publication

- ☐ Peerage of
- ☐ Publons
- ☐ PubMed
- ☐ PubPeer
- ☐ PaperCritic
- ☐ RubriQ
- ☐ Academic
- (and also) others ☐ (and also)

b. What tools/sites do you use to measure impact?

- ☐ JCR (impact
- ☐ Altmetric
- ☐ Scopus
- ☐ ImpactStory
- ☐ PLoS article
- WEB OF SCIENCE
- HARZING.COM
- (and also) others

Web of | Harzing | (and also)

8 Three final general questions about scholarly communication

a. What do you think will be the most important development in scholarly communication in the coming years?

b. Do you support the goal of Open Access?

Yes No I don't know

c. Do you support the goals of Open Science?

(i.e. openly creating, sharing and assessing research, wherever viable)

Yes No I don't know

9 If you would like to receive a summary of your responses, please leave your e-mail address below. You can also indicate whether we can contact you to further discuss how researchers decide which tools to use for scholarly communication. Your e-mail addres

a. Enter your e-mail address here (if you wish)

b. Can we contact you to further discuss scholarly communication workflows?

○ Yes ○ No

Send
