# Supplementary material for: Innovations in scholarly communication - global survey on research tool usage
Source: F1000Res. 2016 Apr 18;5:692. [Version 1] doi: 10.12688/f1000research.8414.1 (PMC4934514; doi:10.12688/f1000research.8414.1)
Supplement: Supplementary file 1 [file f1000research-5-9058-s0000.tgz › 300a78fa-e12e-4789-ab1f-4cb604012e9a.zip!/Surveys/101_Innovations_survey_French.pdf]

1 Démographie & discipline(s) scientifique(s)

a. Quel est votre rôle dans la recherche? *

- Professeur / Professeur associé / Professeur assistant
- Postdoc
- Doctorant
- Étudiant en Licence / Master
- Bibliothécaire / Documentaliste
- Éditeur
- Industrie / Gouvernement
- Autre

b. Quel est le pays de votre affiliation actuelle (ou de la dernière)? *

c. Quelle est votre discipline scientifique? *

- Sciences physiques
- Ingénierie & technologie
- Sciences de la vie
- Médecine
- Sciences sociales & économie
- Droit
- Arts & sciences humaines

d. De quelle année date votre première publication scientifique?

○ Je n'ai pas (encore) publié ○ 2011-2016 ○ 2006-2010 ○ 2001-2005
○ 1991-2000 ○ avant 1991

Dans la partie suivante de l'enquête, nous vous demandons d'identifier les outils/sites que vous utilisez pour vos diverses activités de recherche.

N'hésitez pas à mentionner les outils que vous utilisez et qui sont spécifiques de votre pays ou votre langue.

Vous pouvez toujours **passer** une question qui ne s'applique pas à vous ou si vous ne souhaitez pas y répondre.

Si vous n'êtes pas un chercheur actif (mais un bibliothécaire/documentaliste, un éditeur, un financeur etc.), veuillez indiquer les outils que vous **recommandez**.

---

2 Découverte

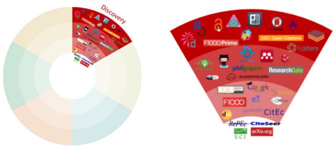

a. Quels outils/sites utilisez-vous pour rechercher de la littérature / des données / etc. ?

- ☐ Google Scholar
- ☐ Web of
- ☐ Scopus
- ☐ Mendeley
- ☐ WorldCat
- ☐ PubMed
- ☐ Paperity
- ☐ (et) autres

b. Quels outils/sites utilisez-vous pour accéder à la littérature etc. ?

- accès par mon institution ou ma bibliothèque — Accès
- Purchase — Achat sur le
- ResearchGate — ResearchGate
- research4life — Research4Life
- Open Access
- deepdyve — Deepdyve
- Envoyer un e-
- (et) autres — (et) autres

c. Quels outils/sites utilisez-vous pour obtenir des alertes / des recommandations ?

- Google scholar — Google Scholar
- Journal TOCs — JournalTOCs
- BrowZine — Browzine
- Mendeley
- F1000Prime — F1000 Prime
- sparrho — Sparrho
- ResearchGate — ResearchGate
- (et) autres — (et) autres

d. Quels outils/sites utilisez-vous pour lire / visionner / annoter ?

consulter la version HTML en ligne

- [ ] Acrobat
- [ ] vue HTML
- [ ] iAnnotate
- [ ] ReadCube
- [ ] UtopiaDocs
- [ ] Mendeley
- [ ] Hypothes.is
- [ ] (et) autres

(et) autres

---

3 Analyse

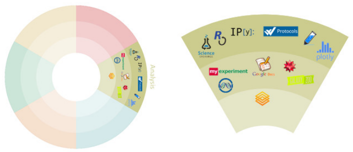

a. Quels outils/sites utilisez-vous pour analyser des données / des textes etc. ?

- [ ] R
- [ ] SPSS
- [ ] Matlab
- [ ] Excel
- [ ] iPython
- [ ] ROpenSci
- [ ] DHbox
- [ ] (et) autres

(et) autres

b. Quels outils/sites utilisez-vous pour partager des notebooks / des protocoles / des processus de travail ?

- ☐ Open Science
- ☐ myExperiment
- ☐ BenchLing
- ☐ Protocols.io
- ☐ Benchfly
- ☐ Scientific
- ☐ Protocol
- (et) autres ☐ (et) autres

---

4 Écriture

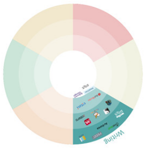

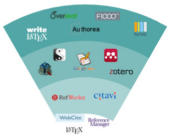

a. Quels outils/sites utilisez-vous pour écrire / préparer votre manuscrit ?

- ☐ Word
- ☐ Google
- ☐ Authorea
- ☐ LaTeX
- ☐ Scrivener
- ☐ Overleaf
- ☐ Scalar
- (et) autres ☐

Overleaf | Scalar | (et) autres

b. Quels outils/sites utilisez-vous pour gérer vos références ?

- [ ] EndNote
- [ ] Zotero
- [ ] RefWorks
- [ ] Mendeley
- [ ] Papers
- [ ] REfME
- [ ] Citavi
- [ ] (et) autres — (et) autres

5 Publication

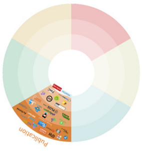

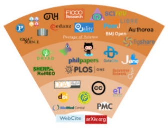

a. Quels outils/sites utilisez-vous pour archiver / partager les publications ?

- [ ] arXiv
- [ ] PubMed
- [ ] Dépôt — Dépôt institutionnel
- [ ] bioRxiv
- [ ] Notes de — Je partage mes notes de travail
- ResearchGate
- SSRN
- (et) autres

- ☐ ResearchGate
- ☐ SSRN
- ☐ (et) autres

b. Quels outils/sites utilisez-vous pour archiver / partager les données et les codes source?

- ☐ GitHub
- ☐ Figshare
- ☐ Zenodo
- ☐ Dryad
- ☐ Dataverse
- ☐ Pangaea
- ☐ BitBucket
- (et) autres ☐ (et) autres

c. Quels outils/sites utilisez-vous pour choisir la revue à laquelle vous allez soumettre votre manuscrit ?

- ☐ JCR (impact
- ☐ DOAJ
- ☐ Scopus
- ☐ Sherpa Romeo
- ☐ QOAM
- SJR
- Journalysis
- (et) autres

d. Quels outils/sites utilisez-vous pour publier ?

- [ ] Revue
- [ ] Revue
- [ ] Megajournal
- [ ] Megajournal
- [ ] Revue de
- [ ] Winnower
- [ ] F1000Research
- [ ] (et) autres

6 Portée

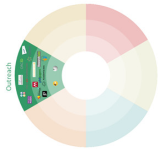

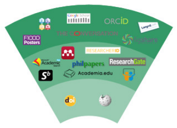

a. Quels outils/sites utilisez-vous pour archiver / partager les posters & les présentations ?

- [ ] Speakerdeck
- [ ] Slideshare
- [ ] F1000Posters
- [ ] ScienceOpen
- [ ] Figshare

Zenodo

Vimeo

(et) autres

(et) autres

b. Quels outils/sites utilisez-vous pour parler de vos recherches en dehors du milieu académique ?

Wikipedia

ResearchBloggi

Wordpress

Kudos

FameLab

Pint of Science

Twitter

(et) autres

(et) autres

c. Quels profils des chercheurs utilisez-vous ?

Google Scholar

ResearchGate

ORCID

Academia.edu

ResearcherID

Page de profil de votre institution	MyScienceWork	(et) autres
☐ Profil sur site	☐ My Science	☐ (et) autres

7 Évaluation

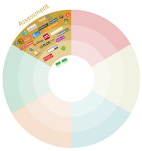

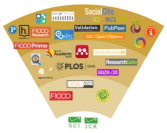

a. Quels outils/sites utilisez-vous pour l'évaluation par les pairs au-delà de ce qui est mis en place par les journaux ?

Il s'agit des commentaires et des évaluations de pairs, pré-publication et post-publication

Peerage of Science	publons	PubMed Commons	PubPeer	PaperCritic
☐ Peerage of	☐ Publons	☐ PubMed	☐ PubPeer	☐ PaperCritic
Rubriq independent peer review system	Academic Karma	(et) autres		
☐ RubriQ	☐ Academic	☐ (et) autres		

b. Quels outils/sites utilisez-vous pour mesurer l'impact ?

JCR (impact

Altmetric

Scopus

ImpactStory

PLoS article

Web of

Harzing

(et) autres

(et) autres

8 Quels sont les outils/sites que vous utilisez qui sont spécifiques de votre pays ou votre langue ?

Sudoc

Francis

HAL

Revues.org

Héloïse

Hypothèses

IdRef

(et) autres

(et) autres

9 Trois questions générales finales sur la communication scientifique

a. Selon vous, quel sera le développement le plus important dans la

communication scientifique au cours des années à venir ?

b. Est-ce que vous soutenez l'objectif du libre accès (Open Access) ?

Oui   Non   Je ne sais pas

c. Est-ce que vous soutenez l'objectif de la science ouverte (Open Science)?

(ce qui signifie la création ouverte, le partage ouvert et l'évaluation ouverte, lorsque c'est possible)

Oui   Non   Je ne sais pas

---

10 Si vous souhaitez recevoir un résumé de vos réponses, veuillez indiquer votre adresse e-mail ci-dessous. Vous pouvez également indiquer si nous pouvons vous contacter pour discuter de la manière dont les chercheurs décident des outils qu'ils util

a. Saisissez votre adresse e-mail ici (si vous le désirez)

b. Pouvons-nous vous contacter pour discuter des processus de communication scientifique ?

Oui   Non

Envoyer
