# Supplementary material for: Innovations in scholarly communication - global survey on research tool usage
Source: F1000Res. 2016 Apr 18;5:692. [Version 1] doi: 10.12688/f1000research.8414.1 (PMC4934514; doi:10.12688/f1000research.8414.1)
Supplement: Supplementary file 1 [file f1000research-5-9058-s0000.tgz › 300a78fa-e12e-4789-ab1f-4cb604012e9a.zip!/Surveys/101_Innovations_survey_Japanese.pdf]

1 あなたの役割・専門分野等

a. 研究におけるあなたの役割は何ですか。 *

- 教授 / 准教授 / 助教授
- 博士課程修了
- 博士課程の学生
- 学士課程/修士課程の学生
- 図書館員
- 出版社
- 産業/政府
- その他

b.

あなたが現在（または近年まで）所属している機関の国はどこですか。 *

c. あなたの専門分野は何ですか。 *

- 物理学
- 工学・技術学
- 生命科学
- 医学
- 社会科学・経済学
- 法学
- 芸術・人文学

d. あなたの最初の学術出版の発行年はいつですか。

- ○ （未だ）発表していない
- ○ 2011年～2016年
- ○ 2006年～2010年
- ○ 2001年～2005年
- ○ 1991年～2000年
- ○ 1991年 より前

次の質問では、あなたが様々な研究活動のためにどのツールやサイトを利用しているかを伺います。

特定の研究活動が該当しない場合、または回答を避ける場合には、質問を飛**ばして**次の質問にお進みいただいて かまいません。

あなた自身が積極的に研究に関わっていない場合には（図書館員、出版社、資金提供者など）、推奨**する**ツールを教えてください。

---

2 文献検索

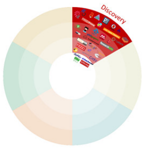

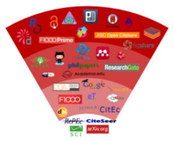

a. 文献やデータなどを探すとき、どのツールまたはサイトを利用しますか。

☐ Google Scholar	☐ Web of	☐ Scopus	☐ Mendeley	☐ WorldCat
☐	☐	（または）その他 ☐		

PubMed | Paperity | （または）そ

b. 文献などにアクセスするとき、どのツールまたはサイトを利用しますか。

- 図書館 / 大学 / 研究機関 からのアクセス — 施設内のアク
- Purchase — 出版社プラッ
- ResearchGate
- Research4Life
- Open Access
- Deepdyve
- 著者にメール
- （または） その他 — （または）そ

c. 通知やレコメンデーションを受ける目的で、どのツールまたはサイトを利用しますか。

- Google Scholar
- JournalTOCs
- Browzine
- Mendeley
- F1000 Prime
- Sparrho
- ResearchGate
- （または） その他 — （または）そ

d. 読む、閲覧する、注釈をつけるために、どのツールまたはサイトを利用しますか。

- Acrobat
- HTML 版をオンラインで閲覧 — HTML 表示を
- iAnnotate
- ReadCube
- UtopiaDocs
- Mendeley
- hypothes.is — Hypothes.is
- （または）その他 — （または）そ

3 分析

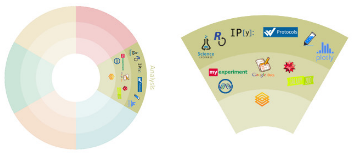

a. データやテキストなどを分析するのに、どのツールまたはサイトを利用しますか。

- R
- SPSS
- Matlab
- Excel
- iPython

- ROpenSci
- DHbox
- （または）その他

（または）そ

b. ノート、 手順/プロトコルまたは業務フロー を共有するのにどのツールまたはサイトを利用しますか。

- Open Science
- myExperiment
- BenchLing
- Protocols.io
- Benchfly
- Scientific
- Protocol
- （または）その他

（または）そ

---

4 執筆

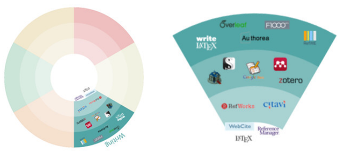

a. 原稿を書くあるいは準備するためにどのツールまたはサイトを利用しますか。

Word | Google | Authorea | LaTeX | Scrivener

Overleaf | Scalar | （または）そ

b. 文献管理にどのツールまたはサイトを利用しますか。

EndNote | Zotero | RefWorks | Mendeley | Papers

REfME | Citavi | （または）そ

5 出版物

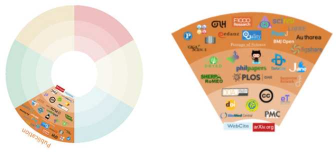

a. 出版物/学術文献の保存または共有にどのツールまたはサイトを利用しますか。

- arXiv.org — arXiv
- PMC — PubMed
- 機関リポジトリ — 機関リポジト
- bioRxiv beta THE PREPRINT SERVER FOR BIOLOGY — bioRxiv
- ワーキングペーパー — 出版前の論文/
- ResearchGate — ResearchGate
- SSRN — SSRN
- (または) その他 — （または）そ

b. データとコードの保存または共有にどのツールまたはサイトを利用しますか。

- GitHub
- figshare — Figshare
- zenodo — Zenodo
- DRYAD — Dryad
- The Dataverse Network™ Project — Dataverse
- PANGAEA — Pangaea
- BitBucket
- (または) その他 — （または）そ

c. どの学術雑誌に原稿を提出するかを決めるためにどのツールまたはサイトを利用しますか。

- JCR (impact
- DOAJ
- Scopus
- Sherpa Romeo
- QOAM
- SCImago
- Journalysis
- （または）その他 — （または）そ

d.
出版する際にどのツールまたはサイトを利用しますか。

- 専門学術誌
- 専門学術誌
- メガジャーナ
- メガジャーナ
- データジャー
- Winnower
- F1000Research
- （または）その他 — （または）そ

6 普及活動

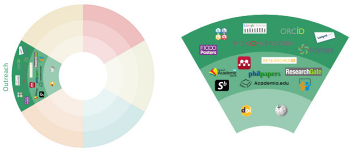

a. ポスターとプレゼンテーションの保存または共有にどのツールまたはサイトを利用しますか。

- [ ] Speakerdeck
- [ ] Slideshare
- [ ] F1000Posters
- [ ] ScienceOpen
- [ ] Figshare
- [ ] Zenodo
- [ ] Vimeo
- [ ] （または）そ

（または）
その他

b. 学究的環境外であなたの研究について発言するのにどのツールまたはサイトを利用しますか。

- [ ] Wikipedia
- [ ] ResearchBloggi
- [ ] Wordpress
- [ ] Kudos
- [ ] FameLab

(または)
その他

Pint of Science | Twitter | （または）そ

c. 研究者のプロフィールの作成と維持のため、どのサイトを使用していますか。

Google Scholar | ResearchGate | ORCID | Academia.edu | ResearcherID

自分の機関の
プロフィール ページ

(または)
その他

自分の機関の | My Science | （または）そ

7 評価

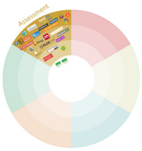

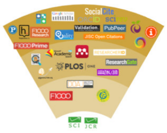

a. 学術雑誌による査読以外で、査読のためにどのツールまたはサイトを利用しますか。

出版前後に得るコメントや査読を意味します。

- [ ] Peerage of
- [ ] Publons
- [ ] PubMed
- [ ] PubPeer
- [ ] PaperCritic
- [ ] RubriQ
- [ ] Academic
- [ ] （または）そ

（または）
その他

b. インパクトを測定するのにどのツールまたはサイトを利用しますか。

- [ ] JCR (impact
- [ ] Altmetric
- [ ] Scopus
- [ ] ImpactStory
- [ ] PLoS article
- [ ] Web of
- [ ] Harzing
- [ ] （または）そ

（または）
その他

---

8 あなたの国または言語を対象とした独自のツールまたはサイトを教えて下さい 。

- [ ] J-STAGE
- [ ] CiNii
- [ ] JAIRO
- [ ] 一太郎
- [ ] エダンズ
- [ ] 研究者リゾル
- [ ] Researchmap
- [ ] （または）そ

（または）
その他

---

9 最後に、学術コミュニケーションに関する三つの一般質問への回答をお願いします。

a. 今後数年の学術コミュニケーションにおける最大の変化は何だと思われますか。

b. オープンアクセスの目的を支持しますか。

○ はい ○ いいえ ○ 分からない

c. オープンサイエンスの目的を支持しますか。

(可能な限り、研究成果をオープンに、共有し、そして評価できるようにする。)

○ はい ○ いいえ ○ 分からない

10 ご自身の回答の要約をお受け取りになりたい場合、以下にメールアドレスをご記入下さい。学術コミュニケーションのためのツールを研究者がどのように決めるかについて私達は調査を進め

a. （よろしければ、）ここにあなたのメールアドレスをご記入下さい。

b. 学術コミュニケーションと研究過程についてお話を聞くために、あなたに直接連絡を差し上げても良いですか。

- ○ はい
- ○ いいえ

送信**する**
