# Supplementary material for: Innovations in scholarly communication - global survey on research tool usage
Source: F1000Res. 2016 Apr 18;5:692. [Version 1] doi: 10.12688/f1000research.8414.1 (PMC4934514; doi:10.12688/f1000research.8414.1)
Supplement: Supplementary file 1 [file f1000research-5-9058-s0000.tgz › 300a78fa-e12e-4789-ab1f-4cb604012e9a.zip!/Surveys/101_Innovations_survey_Russian.pdf]

1 Данные о демографии и научной дисциплине

a. Какова Ваша роль в процессе исследований? *

- ○ Профессор / Доцент
- ○ Старший преподаватель
- ○ Аспирант
- ○ Бакалавр / Магистр
- ○ Библиотекарь
- ○ Издатель
- ○ Промышленность / Правительство
- ○ Другое

b. Страна (в настоящее время или последняя), в которой вы занимаетесь исследованиями? *

c. В сфере каких дисциплин Вы работаете? *

- ☐ Естественные науки
- ☐ Техника и технологии
- ☐ Биологические науки
- ☐ Медицина
- ☐ Общественные науки и экономика
- ☐ Юриспруденция
- ☐ Искусство и гуманитарные науки

d. Каким годом датируется Ваша первая научная публикация?

- ( ) (Пока) нет публикаций
- ( ) 2011-2016
- ( ) 2006-2010
- ( ) 2001-2005
- ( ) 1991-2000
- ( ) До 1991

На следующей странице опроса мы попросим Вас указать средства/сайты, используемые Вами для различных научных исследований.

Можете указать любые средства, которые ориентированы только на Вашу страну или язык.

Можно **пропустить** вопрос, если затронутая в нем область научной деятельности не имеет к Вам отношения или Вы не хотите отвечать на него.

Если Вы не активный исследователь, а библиотекарь, издатель, спонсор и т.д., то укажите средства, которые Вы бы **рекомендовали** к использованию.

---

2 Поиск

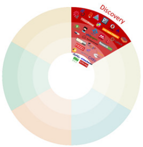

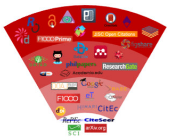

a. Какими средствами/сайтами Вы пользуетесь для поиска литературы/данных / и т.п.?

- [ ] Google Scholar
- [ ] Web of
- [ ] Scopus
- [ ] Mendeley
- [ ] WorldCat
- PubMed

PubMed

Paperity

(а также) другие

(а также)

b. Какими средствами/сайтами Вы пользуетесь для получения доступа к литературе и т.п.?

Доступ через Учреждение или библиотеку

Учрежденческ

Purchase

купить на

ResearchGate

ResearchGate

Research4Life

Open Access

Deepdyve

Запрос по e-

(а также) другие

(а также)

c. Какими средствами/сайтами Вы пользуетесь для получения оповещений / рекомендаций?

Google Scholar

JournalTOCs

Browzine

Mendeley

F1000 Prime

Sparrho

ResearchGate

ResearchGate

(а также) другие

(а также)

d. Какими средствами/сайтами Вы пользуетесь для чтения / просмотра / аннотаций?

Acrobat

просмотр HTML-версии онлайн

просмотр в

iAnnotate

ReadCube

UtopiaDocs

Mendeley

hypothes.is

Hypothes.is

(а также) другие

(а также)

---

3 Анализ

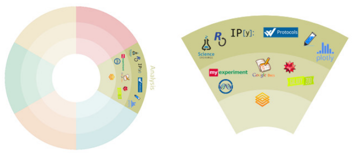

a. Какими средствами/сайтами Вы пользуетесь для анализа данных / текстов и т.п.?

- R
- SPSS
- Matlab
- Excel
- iPython
- ROpenSci
- DHbox
- (а также) другие

b. Какими средствами/сайтами Вы пользуетесь, чтобы делиться записями / протоколами / рабочими процессами?

- Open Science
- myExperiment
- BenchLing
- Protocols.io
- Benchfly
- Scientific
- Protocol
- (а также) другие

4 Написание

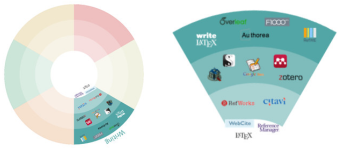

a. Какими средствами/сайтами Вы пользуетесь для написания / подготовки своей рукописи?

- Word
- Google
- Authorea
- LaTeX
- Scrivener
- Overleaf
- Scalar
- (а также) другие — (а также)

b. Какими средствами/сайтами Вы пользуетесь для работы с библиографической информацией?

- EndNote
- Zotero
- RefWorks
- Mendeley
- Papers
- REfME
- Citavi
- (а также) другие — (а также)

5 Публикация

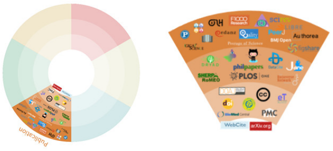

a. Какими средствами/сайтами Вы пользуетесь, чтобы архивировать/ делиться публикациями?

- arXiv.org — arXiv
- PMC — PubMed
- Институцио-нальный репозитарий — Институциона
- bioRxiv beta THE PREPRINT SERVER FOR BIOLOGY — bioRxiv
- Я делюсь Рабочими Документами — Рабочие
- ResearchGate — ResearchGate
- SSRN — SSRN
- (а также) другие — (а также)

b. Какими средствами/сайтами Вы пользуетесь, чтобы архивировать/ делиться данными и программным кодом?

- GitHub
- figshare — Figshare
- zenodo — Zenodo
- DRYAD — Dryad
- The Dataverse Network™ Project — Dataverse
- PANGAEA
- (а также) другие

Pangaea | BitBucket | (а также)

c. Какими средствами/сайтами Вы пользуетесь для выбора журнала, в который отправить свою рукопись?

- JCR (impact
- DOAJ
- Scopus
- Sherpa Romeo
- QOAM
- SCImago
- Journalysis
- (а также) другие — (а также)

d. Какими средствами/сайтами Вы пользуетесь для публикации?

- Тема журнал
- Тема журнал
- Мега-журнал
- Мега-журнал
- Дата-журнал
- Winnower
- F1000Research
- (а также) другие — (а также)

6 Популяризация

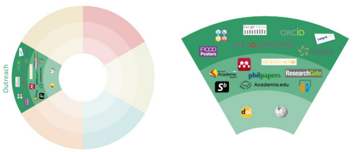

a. Какими средствами/сайтами Вы пользуетесь, чтобы архивировать/ делиться постерами и презентациями?

- Speakerdeck
- Slideshare
- F1000Posters
- ScienceOpen
- Figshare
- Zenodo
- Vimeo
- (а также) другие — (а также)

b. Какими средствами/сайтами Вы пользуетесь, чтобы рассказать о своем исследовании за пределами академической среды?

- Wikipedia
- ResearchBloggi
- Wordpress
- Kudos
- FameLab

(а также) другие

Pint of Science

Twitter

(а также)

c. Какими исследовательскими профилями Вы пользуетесь?

Google Scholar

ResearchGate

ORCID

Academia.edu

ResearcherID

Профильная Страница в своем учреждении

Профильная

My Science

(а также) другие

(а также)

7 Оценка

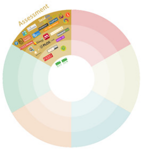

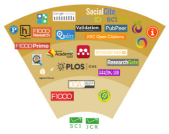

a. Какими средствами/сайтами Вы пользуетесь для получения рецензий, кроме тех, которые предоставляют научные журналы?

Речь идет о комментировании и рецензировании до и после публикации

- [ ] Peerage of
- [ ] Publons
- [ ] PubMed
- [ ] PubPeer
- [ ] PaperCritic
- [ ] RubriQ
- [ ] Academic
- [ ] (а также)

(а также) другие

b. Какими средствами/сайтами Вы пользуетесь для оценки воздействия исследования?

- [ ] JCR (impact
- [ ] Altmetric
- [ ] Scopus
- [ ] ImpactStory
- [ ] PLoS article
- [ ] Web of
- [ ] Harzing
- [ ] (а также)

(а также) другие

---

8 Какие из используемых Вами средств/сайты ориентированы исключительно на Вашу страну или язык?

- [ ] Киберленинка
- [ ] elibrary.ru
- [ ] mathnet.ru
- [ ] БД Винити
- [ ] Соционет
- [ ] ВКонтакте
- [ ] Science Index
- [ ] (а также) другие — (а также)

---

9 Три завершающих общих вопроса о научных коммуникациях

a. Что по Вашему мнению станет наиболее важным достижением в развитии научных коммуникаций в ближайшие годы?

b. Поддерживаете ли Вы цели, преследуемые концепцией Открытого доступа (Open Access)?

○ Да ○ Нет ○ Не знаю

c. Поддерживаете ли Вы цели, преследуемые концепцией Открытой науки (Open Science)?

(т.е. открытое создание, распространение и оценка результатов исследований везде, где это применимо)

○ Да ○ Нет ○ Не знаю

10 Если Вы хотите получить краткий обзор своих ответов, оставьте свой адрес электронной почты ниже.   Вы также можете указать, можем ли мы связ

a. Введите здесь свой адрес электронной почты (по желанию)

b. Можно ли связаться с Вами для дальнейшего обсуждения рабочих процессов в сфере научных коммуникаций?

- Да
- Нет

Отправить
