# Supplementary material for: Innovations in scholarly communication - global survey on research tool usage
Source: F1000Res. 2016 Apr 18;5:692. [Version 1] doi: 10.12688/f1000research.8414.1 (PMC4934514; doi:10.12688/f1000research.8414.1)
Supplement: Supplementary file 1 [file f1000research-5-9058-s0000.tgz › 300a78fa-e12e-4789-ab1f-4cb604012e9a.zip!/Surveys/101_Innovations_survey_Spanish.pdf]

1 Datos demográficos & disciplina(s)

a. ¿Cuál es su rol en la investigación? *

- ○ Profesor / Profesor Asociado / Profesor Asistente
- ○ Investigador postdoctoral
- ○ Estudiante de doctorado
- ○ Estudiante de grado / maestría
- ○ Bibliotecario/a
- ○ Editor
- ○ Industria / Gobierno
- ○ Otro

b. ¿Cuál es el país de su actual (o última) afiliación? *

c. ¿En qué disciplina(s) está trabajando? *

- ☐ Ciencias físicas
- ☐ Ingeniería y tecnología
- ☐ Ciencias de la vida
- ☐ Medicina
- ☐ Ciencias sociales y economía
- ☐ Ciencias jurídicas y derecho
- ☐ Artes y humanidades

d. ¿De qué año data su primera publicación académica?

- [ ] (Todavía) no he publicado
- [ ] 2011-2016
- [ ] 2006-2010
- [ ] 2001-2005
- [ ] 1991-2000
- [ ] Antes de 1991

En la siguiente parte de la encuesta, le pedimos que identifique qué herramientas/sitios utiliza para las distintas actividades de investigación.

No dude en mencionar cualquier herramienta que utiliza que esté orientada solamente a su país o idioma.

Siempre puede **omitir** una pregunta si la actividad de investigación en particular no se aplica a usted o si prefiere no contestar.

Si usted no es un investigador activo (sino un bibliotecario, editor, patrocinador etc.), por favor, indique qué herramientas **recomienda**.

---

2 Descubrir

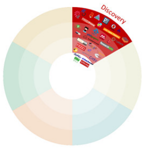

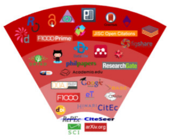

a. ¿Qué herramientas/sitios utiliza para buscar bibliografía / datos / etc.?

- [ ] Google Scholar
- [ ] Web of
- [ ] Scopus
- [ ] Mendeley
- [ ] WorldCat
- [ ] PubMed
- [ ] Paperity
- [ ] (y también) otros — (y también)

b. ¿Qué herramientas/sitios utiliza para tener acceso a bibliografía etc.?

- Acceso a través de mi institución o biblioteca — ☐ Acceso
- Purchase — ☐ Pago en
- ResearchGate — ☐ ResearchGate
- research4life — ☐ Research4Life
- ☐ Open Access
- deepdyve — ☐ Deepdyve
- @ — ☐ Correo
- (y también) otros — ☐ (y también)

c. ¿Qué herramientas/sitios utiliza para recibir alertas / recomendaciones?

- Google scholar — ☐ Google Scholar
- Journal TOCs — ☐ JournalTOCs
- BrowZine — ☐ Browzine
- ☐ Mendeley
- F1000 Prime — ☐ F1000 Prime
- sparrho — ☐ Sparrho
- ResearchGate — ☐ ResearchGate
- (y también) otros — ☐ (y también)

d. ¿Qué herramientas/sitios utiliza para leer / ver / anotar?

Acrobat

Ver versión HTML en línea

Uso la vista

iAnnotate

ReadCube

UtopiaDocs

Mendeley

hypothes.is

Hypothes.is

(y también) otros

(y también)

3 Analizar

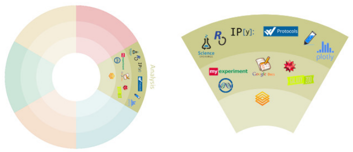

a. ¿Qué herramientas/sitios utiliza para analizar datos / textos etc.?

R

SPSS

Matlab

Excel

iPython

ROpenSci

DHbox

(y también) otros

(y también)

b. ¿Qué herramientas/sitios utiliza para compartir cuadernos de notas / protocolos / flujos de trabajo?

- [ ] Open Science
- [ ] myExperiment
- [ ] BenchLing
- [ ] Protocols.io
- [ ] Benchfly
- [ ] Scientific
- [ ] Protocol
- [ ] (y también) otros — (y también)

---

4 Escribir

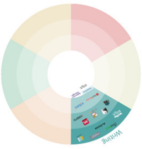

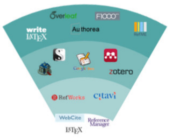

a. ¿Qué herramientas/sitios utiliza para escribir / preparar su manuscrito?

- [ ] Word
- [ ] Google
- [ ] Authorea
- [ ] LaTeX
- [ ] Scrivener
- [ ] Overleaf
- [ ] Scalar
- [ ] (y también) otros

Overleaf | Scalar | (y también)

b. ¿Qué herramientas/sitios utiliza para la gestión de referencias?

- [ ] EndNote
- [ ] Zotero
- [ ] RefWorks
- [ ] Mendeley
- [ ] Papers
- [ ] REfME
- [ ] Citavi
- [ ] (y también) otros — (y también)

5 Publicar

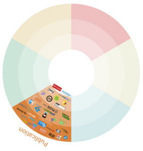

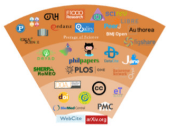

a. ¿Qué herramientas/sitios utiliza para archivar / compartir publicaciones?

- [ ] arXiv
- [ ] PubMed
- [ ] Repositorio institucional — Repositorio
- [ ] bioRxiv
- [ ] Comparto documentos de trabajo — Documentos
- ResearchGate
- SSRN
- (y también) otros

ResearchGate | SSRN | (y también)

b. ¿Qué herramientas/sitios utiliza para archivar/compartir datos y códigos?

GitHub | Figshare | Zenodo | Dryad | Dataverse

Pangaea | BitBucket | (y también) otros (y también)

c. ¿Qué herramientas/sitios utiliza para decidir a qué revista enviar su manuscrito?

JCR (impact | DOAJ | Scopus | Sherpa Romeo | QOAM

(y también) otros

d. ¿Qué herramientas/sitios utiliza para publicar?

- [ ] Revista
- [ ] Revista
- [ ] Megajournal
- [ ] Megajournal
- [ ] Revista de
- [ ] Winnower
- [ ] F1000Research
- [ ] (y también)

(y también) otros

6 Divulgar

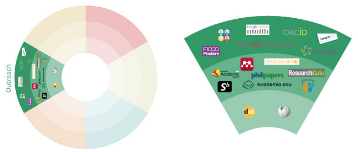

a. ¿Qué herramientas/sitios utiliza para archivar/compartir pósteres y presentaciones?

- [ ] Speakerdeck
- [ ] Slideshare
- [ ] F1000Posters
- [ ] ScienceOpen
- [ ] Figshare

Zenodo

Vimeo

(y también) otros

(y también)

b. ¿Qué herramientas/sitios utiliza para contar de su investigación fuera del ámbito académico?

Wikipedia

ResearchBloggi

Wordpress

Kudos

FameLab

Pint of Science

Twitter

(y también) otros

(y también)

c. ¿Qué perfiles de investigador utiliza?

Google Scholar

ResearchGate

ORCID

Academia.edu

ResearcherID

Página de perfil en su propia institución	MyScienceWork	(y también) otros
☐ Página de perfil	☐ My Science	☐ (y también)

7 Evaluar

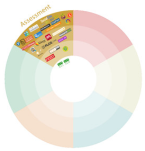

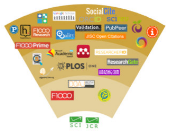

a. ¿Qué herramientas/sitios utiliza para la revisión por pares aparte de las organizadas por revistas?

Se trata de comentarios y revisión por pares, tanto antes como después de la publicación

Peerage of Science	publons	PubMed Commons	PubPeer	PaperCritic
☐ Peerage of	☐ Publons	☐ PubMed	☐ PubPeer	☐ PaperCritic
Rubriq	Academic Karma	(y también) otros		
☐ RubriQ	☐ Academic	☐ (y también)		

b. ¿Qué herramientas/sitios utiliza para medir la repercusión?

- [ ] JCR (impact
- [ ] Altmetric
- [ ] Scopus
- [ ] ImpactStory
- [ ] PLoS article
- [ ] Web of
- [ ] Harzing
- [ ] (y también) otros — (y también)

---

8 ¿Qué herramientas/sitios utiliza que están orientados a su país o idioma?

- [ ] SciELO
- [ ] Redalyc
- [ ] Dialnet
- [ ] Latindex
- [ ] Red de
- [ ] CLACSO
- [ ] MIAR
- [ ] (y también) otros — (y también)

---

9 Tres preguntas generales finales acerca de la comunicación científica

a. ¿Qué cree usted que será el desarollo más importante en la comunicación científica en los próximos años?

b. ¿Apoya usted el objetivo del Acceso Abierto?

○ Sí ○ No ○ No sé

c. ¿Apoya usted el objetivo de la Ciencia Abierta?

(es decir abiertamente la creación, el intercambio y la evaluación de la investigación, siempre que sea viable)

○ Sí ○ No ○ No sé

---

10 Si desea recibir un resumen de sus respuestas, por favor, déjenos a continuación su dirección de correo electrónico. También puede indicar si nos podemos poner en contacto con usted para seguir discutiendo cómo los investigadores deciden qué herra

a. Introduzca aquí su dirección de correo electrónico (si lo desea)

b. ¿Podemos contactarle para seguir compartiendo sobre los flujos de trabajo de la comunicación científica?

○ Sí ○ No

Enviar
