# Supplementary material for: Innovations in scholarly communication - global survey on research tool usage
Source: F1000Res. 2016 Apr 18;5:692. [Version 1] doi: 10.12688/f1000research.8414.1 (PMC4934514; doi:10.12688/f1000research.8414.1)
Supplement: Peer review history

# Peer review: Referee response for version 1

**DOI:** 10.5256/f1000research.9058.r14417

**Published:** 2016-07-01

**Authors:** Isabella Peters, Kaltrina Nuredini

**Affiliations:** 1 Kiel University, Kiel, Germany; 2 ZBW Leibniz Information Centre for Economics, Kiel, Germany

The authors present a data note which aims at describing a data set by giving details on how data was collected and processed and which software or protocols were used, but which will not provide an analysis of the data, results, or conclusions.

 The authors met these F1000Research requirements and, accordingly, describe the setup of the survey, give details on the sampling methods and ways of disseminating the survey request, and briefly introduce the distribution of responses. They also discuss the population, sample size and response rate as well as the completeness of responses and observed and biases in the data. Information on the post-hoc data processing (i.e., anonymization, cleaning, and harmonization of data) is also given. The data note finishes with a quantitative description of the data, how it is stored (i.e. openly on zenodo, as required by F1000Research), and how it can be accessed.

 Overall, the description of the data and the data processing is sound, seems to be reasonable, and as far as I can assess meet the standards of studies of that kind. The data generation is also suitable for investigations of usage of tools and the data set will serve the understanding of scholarly communication in the digital era in general and on social media in particular. Moreover, the data set cannot only answer if researchers use particular tools but also for what purposes or in what steps of the research cycle respectively.

 However, to get a more complete view on how the data has exactly been processed and collected, as well as to enhance repeatability of the study and to aid interpretation of results in later research making use of this data set I recommend adding information to following questions (which mostly refer to initial premises set by the authors of the survey and which have to been known in order to comprehend the processing steps that have been taken):

1. Activities were selected from a database developed by the authors. How did you create the database – how did you find the entries? Could tool providers register themselves? How complete is it?
2. Described activities in the survey were chosen for their overall importance/ the most well-known tools were selected as answers: How do you define “overall importance” and “most well-known”? How did you determine this selection? Can you provide evidence (even if this is a data note)? Have you taken into account disciplinary peculiarities?
3. Was it possible to choose more than one tool as answer in the survey (adding up to answer numbers >100%)?
4. Was it possible for participants to answer the survey more than once? Have you detected any bot-like behavior?
5. Six obvious spam answers have been removed from the data set: can you give examples on what was considered spam?

We have read this submission. We believe that we have an appropriate level of expertise to confirm that it is of an acceptable scientific standard.

# Peer review: Referee response for version 1

**DOI:** 10.5256/f1000research.9058.r14542

**Published:** 2016-06-28

**Authors:** Samuel Illingworth

**Affiliations:** 1 School of Research, Enterprise & Innovation, Manchester Metropolitan University, Manchester, UK

This is an exceptionally well-designed survey, which was carried out professionally and effectively. The results of this survey will be incredibly useful for future researchers who want to gain an insight into current practices relating to innovations in scholarly communications. 

 The transparency of this data set, both upon completion and also throughout the collection period is commendable and is something that I would like to hold up as an example of best practice. The authors have worked tirelessly to ensure that this data set is of the greatest possible value to the wider research community. In particular, the use of the WordPress blog and the presentation of the final data set on Silk are processes that I would like to see repeated by others.

 I have only a couple of queries relating to the survey's design and implementation:

1. What quota sampling strategy was used? In the **Distribution of the survey; sampling ** section the authors mention that quota sampling was used, but how was this done, which quotas were selected, and why were they chosen?
2. In the **Translation of the survey** section the authors mention that the Arabic survey did not include "one additional question at the end of the survey on the use of tools targeting that specific language area," why was this the case?

Apart from these two small details, I would like to commend the authors on such an excellent dataset, which sets a very high standard from research design right through to dissemination of results. I am also very much looking forward to what future analysis of the data will reveal about current practices relating to innovations in scholarly communications.

I have read this submission. I believe that I have an appropriate level of expertise to confirm that it is of an acceptable scientific standard.
